# Validation of podocalyxin-like protein as a biomarker of poor prognosis in colorectal cancer

**DOI:** 10.1186/1471-2407-12-282

**Published:** 2012-07-08

**Authors:** Anna Larsson, Marie Fridberg, Alexander Gaber, Björn Nodin, Per Levéen, Göran Jönsson, Mathias Uhlén, Helgi Birgisson, Karin Jirström

**Affiliations:** 1 Department of Clinical Sciences, Division of Pathology, Lund University, Skåne University Hospital, 221 85, Lund, Sweden; 2 Department of Clinical Sciences, Division of Oncology, Lund University, Skåne University Hospital, 221 85, Lund, Sweden; 3 Department of Biotechnology, AlbaNova University Center, Royal Institute of Technology, 106 91, Stockholm, Sweden; 4 Department of Surgical Sciences, Colorectal Surgery, Uppsala University, 751 85, Uppsala, Sweden

## Abstract

**Background:**

Podocalyxin-like 1 (PODXL) is a cell-adhesion glycoprotein and stem cell marker that has been associated with an aggressive tumour phenotype and adverse outcome in several cancer types. We recently demonstrated that overexpression of PODXL is an independent factor of poor prognosis in colorectal cancer (CRC). The aim of this study was to validate these results in two additional independent patient cohorts and to examine the correlation between PODXL mRNA and protein levels in a subset of tumours.

**Method:**

PODXL protein expression was analyzed by immunohistochemistry in tissue microarrays with tumour samples from a consecutive, retrospective cohort of 270 CRC patients (cohort 1) and a prospective cohort of 337 CRC patients (cohort 2). The expression of PODXL mRNA was measured by real-time quantitative PCR in a subgroup of 62 patients from cohort 2. Spearman´;s Rho and Chi-Square tests were used for analysis of correlations between PODXL expression and clinicopathological parameters. Kaplan Meier analysis and Cox proportional hazards modelling were applied to assess the relationship between PODXL expression and time to recurrence (TTR), disease free survival (DFS) and overall survival (OS).

**Results:**

High PODXL protein expression was significantly associated with unfavourable clinicopathological characteristics in both cohorts. In cohort 1, high PODXL expression was associated with a significantly shorter 5-year OS in both univariable (HR = 2.28; 95% CI 1.43-3.63, p = 0.001) and multivariable analysis (HR = 2.07; 95% CI 1.25-3.43, p = 0.005). In cohort 2, high PODXL expression was associated with a shorter TTR (HR = 2.93; 95% CI 1.26-6.82, p = 0.013) and DFS (HR = 2.44; 95% CI 1.32-4.54, p = 0.005), remaining significant in multivariable analysis, HR = 2.50; 95% CI 1.05-5.96, p = 0.038 for TTR and HR = 2.11; 95% CI 1.13-3.94, p = 0.019 for DFS.

No significant correlation could be found between mRNA levels and protein expression of PODXL and there was no association between mRNA levels and clinicopathological parameters or survival.

**Conclusions:**

Here, we have validated the previously demonstrated association between immunohistochemical expression of PODXL and poor prognosis in CRC in two additional independent patient cohorts. The results further underline the potential utility of PODXL as a biomarker for more precise prognostication and treatment stratification of CRC patients.

## Background

CRC is the third most common type of cancer in the world with an annual worldwide incidence of more than one million cases [1]. Early detection, adequate surgical excision and optimal use of adjuvant treatment are of critical importance for the clinical outcome. Currently, tumour stage at diagnosis is the most important prognostic factor and adjuvant chemotherapy is recommended for all patients with stage III disease to reduce the relative risk of recurrence with approximately 30%.

The role of adjuvant chemotherapy in stage II disease is more unclear, and there is an ongoing debate on how to identify patients with high-risk disease who have a greater risk of recurrence and might benefit from adjuvant therapy. According to ASCO guidelines, adjuvant treatment should be considered for patients with stage II disease presenting with one or more risk factors (including large tumour, tumour perforation, vascular or neural invasion and insufficient lymph node staging) [2][3]. Thus, there is a great need for new prognostic and treatment predictive biomarkers to select patients with high-risk disease, but despite many efforts no well-validated molecular markers have yet been incorporated into clinical pratice.

Podocalyxin-like 1 (PODXL) is an anti-adhesive transmembrane protein belonging to the CD34 family. PODXL inhibits cell-cell interaction through charge-repulsive effects and in normal tissues, its presence has classically been ascribed to hematopoetic progenitor cells [4], vascular endothelial cells [5] and renal glomerular podocytes where it plays a vital part in maintaining filtration pathways [6]. Loss of PODXL expression has been observed in glomerulopathies primarily associated with the nephrotic syndrome [7]. PODXL has been associated with an aggressive tumour phenotype and adverse outcome in several cancer types [8][9,10]. The mechanisms behind these observations are not fully known, but PODXL has been shown to interact with mediators of metastasis [9,11] and to play an important role in epithelial-mesenchymal transition (EMT) [12]. A recent study demonstrated that forced PODXL expression in ovarian cancer cells decreased their adhesivity by altering β1-integrin levels, and that PODXL expression on the cell surface was associated with poor prognosis in high grade serous carcinomas [13]. PODXL has also been demonstrated as being a target of tumour suppressive miRNA-199 in testicular cancer [14].

In a previous study, we have demonstrated that membranous expression of PODXL is associated with unfavourable clinicopathological characteristics and independently predicts a poor prognosis in CRC [15]. The aim of this study was to validate these results in two additional independent patient cohorts with a total number of 590 CRC cases. A secondary aim was to examine the correlation between PODXL mRNA and protein levels and its clinical significance in a subset of the tumours.

## Methods

### Patients

Cohort 1 is a consecutive, retrospective cohort comprising all patients who underwent surgery for CRC at Skåne University Hospital in Malmö, Sweden between 1 January 1990 and 31 December 1991, for whom archival tumour tissue was available (n = 270). The cohort has been described in detail previously [16].

Cohort 2 consisted of 337 patients undergoing surgery for CRC at the Central District Hospital in Västerås, Sweden between June 2000 and December 2003. Tumour tissue for tissue microarray (TMA) construction was available from 320 (95%) patients. Follow-up started at date of diagnosis and ended at death or 15 April 2010 [16][17]. Endpoints were defined according to Punt et al [18]. All observations were censored at loss to follow-up and at the end of the study period.

Information on vital status and cause of death was obtained from the Regional Oncology Registry and hospital records. Histopathological, clinical and treatment data were obtained from pathology and hospital records. Information on recurrence, cause-specific survival and adjuvant therapy was not available for cohort 1.

Patient and tumour characteristics are summarized in Table 1.

**Table 1:** Association between PODXL protein expression and clinicopathological parameters

	**Protein expression cohort 1**			**Protein expression cohort 2**		
n (%)	low 235(90.4)	high 25(9.6)	p-value	low 291(92.1)	high 25(7.9)	p-value
Age						
<=75	130(55.3)	16(64.0)	0.406	166(57.0)	137(69.9)	0.625
>75	105(44.7)	9(36.0)		125(43.0)	59(30.1)	
Gender						
Female	116(52.2)	14(65.2)	0.528	143(49.1)	13(52.0)	0.784
Male	119(47.8)	11(34.8)		148(50.9)	12(48.0)	
T stage						
1, 2	78(33.2)	8(32.0)	0.979	52(17.9)	1(4.0)	0.017
3	124(52.8)	14(56.0)		193(66.3)	15(60.0)	
4	28(11.9)	3(12.0)		46(15.8)	9(36.0)	
Missing	5(2.1)					
N stage						
0	154(65.5)	9(36.0)	0.005	179(61.7)	5(20.0)	<0.001
1	53(22.6)	8(32.0)		57(19.7)	8(32.0)	
2	23(9.8)	7(28.0)		54(18.6)	12(48.0)	
Missing	5 (2.1)	1 (4.00)				
M stage						
0	201(85.5)	19(87.0)	0.150	262(90.0)	14(56.0)	<0.001
1	31(13.2)	6(13.0)		29(10.0)	11(44.0)	
Missing	3(1.3)					
Location						
Colon	189(78.3)	22(78.3)	0.376	194(66.7)	16(64.0)	0.057
Rectum	45(21.7)	3(21.7)		97 (33.3)	9(36.0)	
Missing	1					
Differentiation grade						
High-intermediate	181(80.4)	13(78.3)	0.006	233(80.1)	15(60.0)	0.019
Low	54(19.6)	12(21.7)		58(19.9)	10(40.0)	
Vascular invasion						
Absent	126(53.6)	6(95.7)	0.003	258(88.7)	18(72.0)	0.016
Present	101(43.0)	19(4.3)		33(11.3)	7(28.0)	
Missing	8(3.4)					
Neural invasion						
Absent	N/A	N/A		286(98.3)	22(88)	0.002
Present				5(1.7)	3(12)	

N/A – not analyzed

χ^2^-test of association was used for 2x2 tables and χ^2^-test for linear trend for tables with more than two rows and/or columns. The number of cases with missing data is given for some variables, but these are not included in the analyses.

Approvals for the present study was obtained from the Ethics Committees at Lund University (ref. 447/2007) and Uppsala University (00/001).

### Tissue microarray construction

Areas representative of cancer were marked on haematoxylin and eosin stained slides and TMAs were constructed as previously described [19]. In brief, two 1.0 mm cores were taken from each tumour and mounted in a new recipient block using a semi-automated arraying device (TMArrayer, Pathology Devices, Westminster, MD, USA). Non-necrotic tumour areas were avoided and, when possible, one core was taken from the centre and periphery of the tumour, respectively.

### Immunohistochemistry and antibody validation

For immunohistochemical analysis, 4 μm TMA-sections were automatically pre-treated using the PT-link system (DAKO, Glostrup, Denmark) and then stained in an Autostainer Plus (DAKO, Glostrup, Denmark) with the affinity-purified polyclonal anti-PODXL antibody HPA 2110 (Atlas Antibodies, Stockholm, Sweden, diluted 1:250). The specificity of this antibody, originally generated within the Human Protein Atlas (HPA) project, has been validated using Western blotting and protein arrays and PODXL protein expression has been mapped by IHC in 48 types of normal tissues and 20 common cancers [20,21] ( http://www.proteinatlas.org). The same antibody was used to detect PODXL expression in CRC in our previous study [15] and in the study on testicular cancer by Cheung et al. [14].

### Evaluation of PODXL staining

PODXL expression was recorded as negative (0), weak cytoplasmic positivity in any proportion of cells (1), moderate cytoplasmic positivity in any proportion (2), distinct membranous positivity in ≤50% of cells (3) and distinct membranous positivity in >50% of cells (4) as previously described [15]. The staining was evaluated by two independent observers (AL and KJ) who were blinded to clinical and outcome data. Scoring differences were discussed in order to reach consensus.

### Real-time quantitative PCR

PODXL mRNA expression was analyzed in 62 fresh frozen tumours from patients in cohort 2. Total RNA isolation (RNeasy, Qiagen, Hilden, Germany), cDNA synthesis (Reverse Transcriptase kit, Applied Biosystems, Warrington, UK) and real-time quantitative PCR (qRT-PCR) analysis with SYBR Green PCR master mix (Applied Biosystems) were performed. Quantification of expression levels was calculated by using the comparative Ct method, normalization according to housekeeping gene PMM1 (forward primer: 5’-GCAAAGGGCTGAGGTTCTC-3’, reverse primer: 5’-TCCCACCAGGGCTAGTCTC-3’) [22]. For PODXL amplification, forward primer with sequence 5’-GTAACTGGGCAAAGTGTG-3’ and reverse primer with sequense 5’-CCTGCACAGAGGGTGTTT-3’ were used. All primers were designed using OligoPerfect Designer (Invitrogen).

### Statistical analysis

For statistical purposes, categories of PODXL protein expression were dichotomized into low (0–2) and high (3–4) based on PODXL staining as previously described [15], and mRNA levels into low and high according to the mean value. Spearman´;s Rho and Chi-square tests were used for comparison of PODXL expression and relevant clinicopathological characteristics. Kaplan-Meier analysis and log rank test were used to illustrate differences in TTR, DFS and 5-year OS according to PODXL mRNA and protein expression. Cox regression proportional hazards models were used for estimation of hazard ratios (HR) for DFS and TTR according to PODXL expression in both uni- and multivariable analysis adjusted for age, gender, TNM status, differentiation grade, neural and vascular invasion. A backward conditional selection method was used for variable selection by the model. All tests were two-sided. A p-value of 0.05 was considered significant. All statistical analyses were performed using SPSS version 19 (SPSS Inc, Chicago, IL).

## Results

### PODXL protein expression

**Figure 1:**
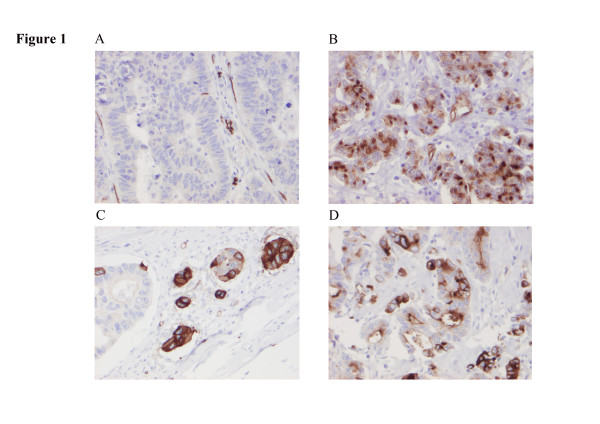
**Immunohistochemical images representing various PODXL staining patterns.** Immunohistochemical images of PODXL staining representing colorectal tumours with (**A**) negative tumour cells and positive stromal vessels, (**B**) moderate cytoplasmic staining without membranous staining and (**C**,**D**) strong, membranous staining in a varying proportion of tumour cells.

Following antibody optimization and staining, PODXL expression could be evaluated in 260/270 (96.3%) tumours in cohort 1 and 316/320 (98.8%) tumours in cohort 2. In cohort 1, 137 (52.7%) tumours were negative for PODXL (score 0), 98 (37.7%) tumours displayed weak-moderate staining (score 1–2) and 25 (9.6%) tumours displayed high PODXL expression (score 3–4). In cohort 2, PODXL expression was denoted as negative (score 0) in 198 (62.7%) tumours, weak-moderate (score 1–2) in 93 (29.5%) tumours and strong (score 3–4) in 25 (7.9%) tumours. Representative IHC images visualizing different staining categories are shown in Figure 1.

### Association between PODXL protein expression and clinicopathological parameters

As shown in Table 1, high PODXL protein expression was associated with more advanced N-stage (p = 0.005), low differentiation grade (p = 0.006) and vascular invasion (p = 0.003) in cohort 1 and with a more advanced T-stage (p = 0.017), N-stage (p < 0.001), M-stage (p < 0.001), low differentiation grade (p = 0.019) and presence of vascular (p = 0.016) and neural invasion (p = 0.002) in cohort 2. There was no significant correlation between PODXL expression and age at diagnosis, gender or tumour location in either of the cohorts.

### Overexpression of PODXL protein is associated with shorter survival and time to recurrence

**Figure 2:**
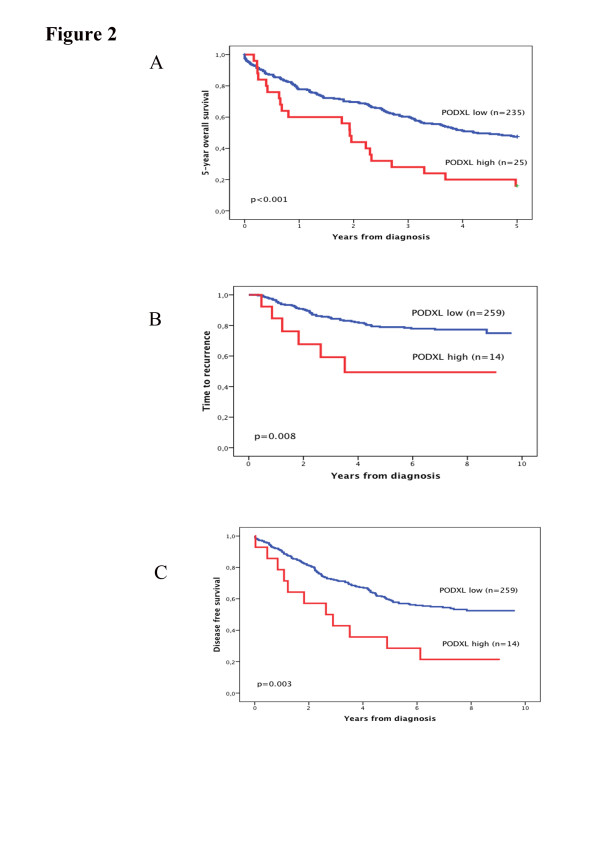
**High expression of PODXL protein is associated with a poor outcome in colorectal cancer patients.** Kaplan-Meier analyses of (**A**) 5-year OS in cohort 1, (**B**) TTR and (C) DFS in cohort 2 according to PODXL expression.

Kaplan Meier analysis demonstrated that high PODXL protein expression correlated with a significantly worse 5-year OS in cohort 1 (p < 0.001) (Figure 2). These associations were confirmed in Cox univariable analysis (HR = 2.28; 95% CI 1.43-3.63, p = 0.001) and remained significant in multivariable analysis adjusted for age, gender, TNM status, differentiation grade and vascular invasion (HR = 2.07; 95% CI 1.25-3.43, p = 0.005). In cohort 2, high PODXL expression was significantly associated with a shorter TTR and DFS in curatively treated patients (Figure 2). Cox univariable analysis confirmed this association with a shorter TTR (HR = 2.93; 95% CI 1.26-6.82, p = 0.013) and DFS (HR = 2.44; 95% CI 1.32-4.54, p = 0.005) (Table 2), remaining significant in multivariable analysis adjusted for age, gender, T- and N- status, differentiation grade, vascular and neural invasion, HR = 2.50; 95% CI 1.05-5.96, p = 0.038 for TTR and HR = 2.11; 95% CI 1.13-3.94, p = 0.019 for DFS (Table 2).

**Table 2:** Cox uni- and multivariable analysis of relative risks of time to recurrence and disease free survival in curatively treated patients from cohort 2 according to PODXL protein expression

	**Time to recurrence**			**Disease free survival**		
	**HR(95%CI)**	***p-value***	**n(events)**	**HR(95%CI)**	***p-value***	**n(events)**
		*Univariable*			*Univariable*	
PODXL low	1,00		259(54)	1,00		259(118)
PODXL high	2.93(1.26-6.82)	0.013	14(6)	2.44(1.32-4.54)	0.005	14(11)
		*Multivariable*			*Multivariable*	
PODXL low	1,00		259(53)	1,00		258(117)
PODXL high	2.50(1,05-5.96)	0.038	14(6)	2.11(1.13-3.94)	0.019	14(11)

Multivariable analysis adjusted for age (>/<=75 years), gender, T-stage (I, II, III, IV), N stage (0,1,2), differentiation grade (high-intermediate, low), neural and vascular invasion (absent, present).

There was no significant difference in outcome, neither for TTR, DFS or OS, between patients with tumours denoted as having negative, weak or moderate staining; i.e. all categories lacking membranous staining had a similar prognosis (data not shown).

### Correlation beween PODXL mRNA levels and protein expression, clinicopathological characteristics and outcome

**Figure 3:**
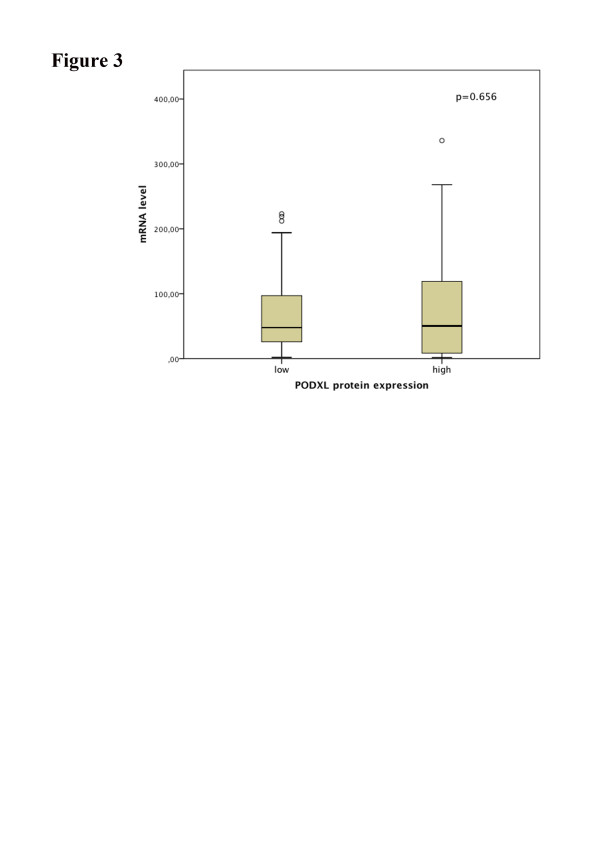
**Association between PODXL gene and protein expression in a subgroup of colorectal patients from cohort 2.** Boxplots of PODXL mRNA levels according to protein expression (low-high). Horizontal lines indicate median.

PODXL mRNA levels were assessed in 62 fresh frozen tumour specimens from cohort 2, comprising 21 tumours with high protein expression and 41 tumours with low protein expression. As visualized in Figure 3, there was no significant correlation between PODXL mRNA levels and protein expression (p = 0.656). Moreover, there was no significant association between PODXL mRNA levels and clinicopathological parameters (Additional file 1) or TTR, DFS and 5-year OS (data not shown). mRNA levels did not differ significantly between tumours lacking PODXL expression compared with categories of any degree of expression (data not shown).

### Correlation between PODXL expression and response to adjuvant chemotherapy

As it has been previously demonstrated that patients with PODXL high tumours might benefit from adjuvant chemotherapy [15] we also investigated the prognostic value of PODXL in combined strata according to chemotherapy. The results revealed similar, however non-significant, trends to the previous study, indicating that patients with PODXL high tumours who were treated with adjuvant chemotherapy had a similar DFS as patients with low PODXL expressing tumours (Additional File 2).

## Discussion

The results from this validation study provide further evidence that overexpression of PODXL protein is a predictor of poor prognosis in CRC, independent of tumour stage or other clinicopathological characteristics. Together with the results from our previous study [15], this correlation has now been confirmed in three independent cohorts representing more than 1000 patients in total. Moreover, while the results from this study further confirm the association of high PODXL expression with a reduced overall survival, its impact as a biomarker of reduced time to recurrence and disease free survival in curatively treated patients is also demonstrated.

While PODXL is expressed in the cytoplasm in a considerable proportion of CRC tumours, it is mainly the presence of distinct membranous staining, here denoted as high expression, that seems to confer a poor prognosis [15]. The most aggressive tumours show a strong, membranous staining in a subset of tumour cells at the invasive tumour front, corresponding well to the morphological term “tumour budding”, which has been demonstrated to be of prognostic importance in CRC [23,24], and biologically closely related to EMT [23][12]. These observations are also well in line with the study by Cipollone et al, where PODXL expression on the cell surface but not in the cytoplasm was significantly associated with a shorter disease-free survival in patients with high grade serous ovarian carcinoma [13]. In that study, it was also demonstrated that forced expression of PODXL in serous ovarian carcinoma-derived OVCAR-3 cells resulted in localization of PODXL to the cell surface, decreased cell adhesion to mesothelial monolayers and diminished levels of β1-integrin, leading the authors to conclude that PODXL may facilitate transperitoneal metastasis of high grade serous carcinoma [13]. In light of the significant association of high PODXL expression and increased T-stage, in particular stage T4 tumours, observed in our previous study [15], and in Cohort II in this study, it would be of interest to perform further studies to investigate whether PODXL may have a role in the initiation of serosal invasion also in CRC.

Since membranous staining of PODXL in most cases is seen in only a fraction of tumour cells, these tumours do not necessarily have the highest level of protein in total and overexpression of protein will not be reflected in high mRNA levels. Hence, evaluation of PODXL expression should be based on a qualitative rather than quantitative assessment and IHC has several advantages over other types of assays in terms of clinical applicability as it is a comparatively simple, fast and inexpensive method.

Considering the prognostic importance of protein localization, the lack of a significant correlation between PODXL mRNA and protein expression levels, clinicopathological characteristics and survival is not surprising. Moreover, being a CD34-related protein, PODXL is expressed on vascular surfaces [25], and thus, present in various amounts in the tumour-associated stroma.

It should also be pointed out that previous studies attempting to determine a direct correlation between mRNA levels and protein expression in tumours have shown divergent results, and analyses indicate that protein concentrations correlate with the corresponding mRNA levels by only 20-40 % [26,27]. In some cases, such as HER2/neu, expression levels show highly significant correlation [28], but in other studies regarding molecular markers in adenocarcinoma of the lung [29] and in prostate cancer [30], a relationship between mRNA and protein was not observed. There are several possible explanations for these discrepancies, e.g. post-translational modifications, variations in protein half-life and actual biological differences between mRNA and protein abundance. In addition, there are potential experimental errors to be considered, including anatomical origin and quality of tissue. A more comprehensive analysis of the correlation between PODXL mRNA and protein expression might be provided by performing microdissection of strongly staining areas from frozen tumour sections. In the clinical setting, however, it is evident that immunohistochemical assessment of PODXL is the method of choice, whereby recognition of its location on the cellular surface should be quite straightforward.

Some limitations related to the TMA-technique must be considered, not least its ability to accurately reflect the expression of heterogenously expressed markers. One way to compensate for this is to, whenever possible, ensure that tumour cores are sampled from different tumour areas, i.e. the invasive front and centre, respectively. While this had been done for the majority of the here analyzed tumours, it should be pointed out that tumour areas denoted as having distinct membranous PODXL expression could not only be found at the invasive front, but also in scattered areas within the tumour. However, we have previously compared results from paired TMA-cores with full-face sections with excellent concordance [15]. Moreover, assessment of full-face sections from prospectively collected clinical samples have reveled a similar proportion of CRC cases with high PODXL expression as reported here and in our previous study (unpublished observations).

In both cohorts in this study, the proportion of tumours denoted as having high PODXL expression was lower compared to our previous study, where 13.4% of the tumours displayed high PODXL expression [15]. Given the observed association between PODXL expression and a more advanced disease stage, this can be explained by the fact that the two cohorts examined here had a lower percentage of patients with stage IV disease (9.6 vs 7.9%) compared to the population-based prospective cohort used in the previous study (18.3%).

Furthermore, in patients with stage III disease in cohort 2, a trend towards the previously demonstrated benefit from adjuvant chemotherapy could be observed for patients with high tumour-specific PODXL expression, who had a similar DFS and OS as patients with PODXL-low tumours [15]. Although this did not reach statistical significance, most likely due to the smaller subgroup available for analysis, these findings further indicate that patients with high PODXL-expressing tumours might benefit from adjuvant treatment. As adjuvant chemotherapy is given to the majority of patients with stage III disease according to current treatment protocols, assessment of PODXL expression might be particularly relevant in order to identify high-risk patients with stage II disease. Similar to our previous study [15] the number of patients with stage II disease in this study who received adjuvant treatment were too few for a meaningful statistical analysis. Hence, these associations should be confirmed in larger retrospective studies or within randomized treatment trials.

## Conclusions

In conclusion, we have validated the previously demonstrated association between immunohistochemical expression of PODXL, defined as distinct membranous staining, and poor prognosis in CRC. The results further underline the potential utility of PODXL as a biomarker for more precise prognostication and treatment stratification of CRC patients. In light of the prognostic relevance of the subcellular localization of PODXL and the observed lack of a significant correlation between PODXL mRNA levels and protein expression, it is evident that IHC remains the most suitable method for assessment of PODXL protein expression in the clinical setting.

## Competing interests

A patent has been filed related to the use of PODXL as a prognostic biomarker in CRC.

## Authors' contributions

AL performed statistical analysis, carried out the functional studies and drafted the manuscript. BN constructed the TMAs. MF carried out the qPCR. PL helped with technical assistance. AG participated in the data collection and helped with statistical analysis. GJ helped with advice on mRNA analysis. MU participated in the design of the study and technical assistance. HB assisted with data collection. KJ conceived of the study and participated in its design and coordination and helped to draft the manuscript. All authors read and approved the final manuscript.

## Pre-publication history

The pre-publication history for this paper can be accessed here:

http://www.biomedcentral.com/1471-2407/12/282/prepub

## Supplementary Material

Additional file 1 Association between PODXL mRNA expression and clinicopathological parameters. Click here for file

Additional file 2 **Survival according to PODXL expression and adjuvant chemotherapy in patients with stage III disease (cohort 2) Kaplan-Meier estimates of (A) DFS and (B) OS according to combinations of PODXL expression (high or low) and adjuvant chemotherapy (CT).** p values correspond to pairwise comparisons of PODXL high and untreated tumours with the other strata, respectively. Click here for file
