# Supplementary figures and images for: Validation of podocalyxin-like protein as a biomarker of poor prognosis in colorectal cancer
Source: BMC Cancer. 2012 Jul 8;12:282. doi: 10.1186/1471-2407-12-282 (PMC3492217; doi:10.1186/1471-2407-12-282)

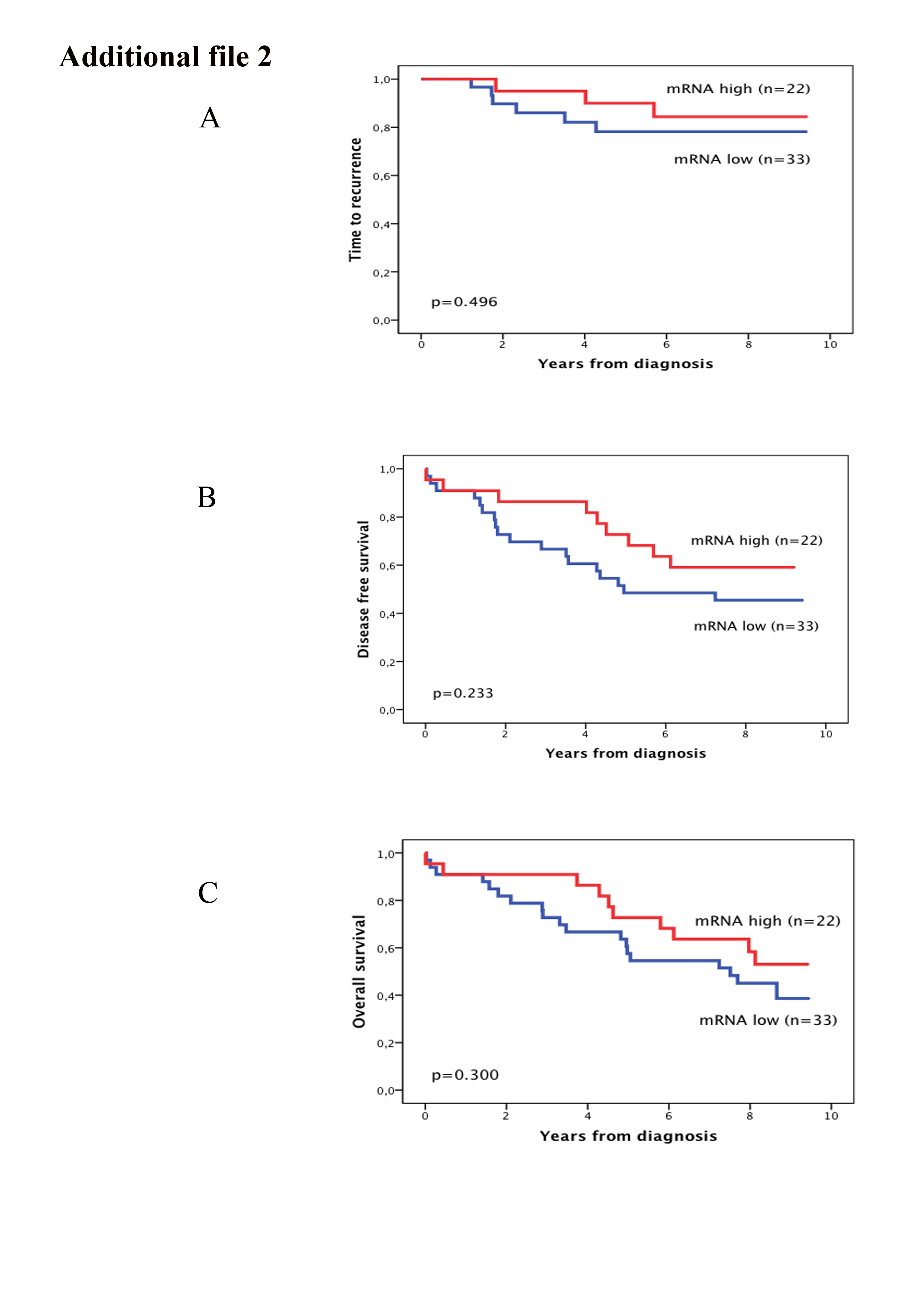

Supplement: Additional file 2 — Survival according to PODXL expression and adjuvant chemotherapy in patients with stage III disease (cohort 2) Kaplan-Meier estimates of (A) DFS and (B) OS according to combinations of PODXL expression (high or low) and adjuvant chemotherapy (CT). p values correspond to pairwise comparisons of PODXL high and untreated tumours with the other strata, respectively. [file 1471-2407-12-282-S2.jpeg]
